# Neuroinvasive West Nile Infection with an Unusual Clinical Presentation: A Single-Center Case Series

**DOI:** 10.3390/tropicalmed5030138

**Published:** 2020-08-31

**Authors:** Nadia Castaldo, Elena Graziano, Maddalena Peghin, Tolinda Gallo, Pierlanfranco D’Agaro, Assunta Sartor, Tiziana Bove, Roberto Cocconi, Giovanni Merlino, Matteo Bassetti

**Affiliations:** 1 Infectious Diseases Division, Department of Medicine, University of Udine and Azienda Sanitaria Universitaria Integrata di Udine, 33100 Udine, Italy; nadiacastaldo.nc@gmail.com (N.C.); elenagraziano22@gmail.com (E.G.); assunta.sartor@asufc.sanita.fvg.it (A.S.); Matteo.Bassetti@hsanmartino.it (M.B.); 2 Department of Prevention, Local Health Unit 4 Medio Friuli, 33100 Udine, Italy; linda.gallo@asufc.sanita.fvg.it; 3 Department of Medical, Surgical and Health Sciences, University of Trieste, 34100 Trieste, Italy; dagaro@burlo.trieste.it; 4 Anesthesiology and Intensive Care Medicine, Department of Medicine, University of Udine and Azienda Sanitaria Universitaria Integrata di Udine, 33100 Udine, Italy; tiziana.bove@uniud.it; 5 SOC Direzione Medica di Presidio, Azienda Sanitaria Universitaria Integrata di Udine, 33100 Udine, Italy; roberto.cocconi@asufc.sanita.fvg.it; 6 Clinical Neurology, University of Udine Medical School, 33100 Udine, Italy; giovanni.merlino@asufc.sanita.fvg.it; 7 Infectious Diseases Unit, Ospedale Policlinico San Martino-IRCCS, 16132 Genoa, Italy

**Keywords:** West Nile Virus, mosquitos, vector, Flavivirus, artropodes, neuroinvasiveness

## Abstract

The 2018 West Nile Virus (WNV) season in Europe was characterized by an extremely high infection rate and an exceptionally higher burden when compared to previous seasons. Overall, there was a 10.9-fold increase in incidence in Italy, with 577 human cases, 230 WNV neuroinvasive diseases (WNNV) and 42 WNV-attributed deaths. Methods: in this paper we retrospectively reported the neurological presentation of 7 patients admitted to University Hospital of Udine with a diagnosis of WNNV, especially focusing on two patients who presented with atypical severe brain stem involvement. Conclusions: the atypical features of some of these forms highlight the necessity to stay vigilant and suspect the diagnosis when confronted with neurological symptoms. We strongly encourage clinicians to consider WNNV in patients presenting with unexplained neurological symptoms in mild climate-areas at risk.

## 1. Background

West Nile Virus (WNV) is a positive stranded RNA Flavivirus. It has an enzoonotic cycle among birds and mosquitos, and can be rapidly spread to several hosts, including humans. WNV infection in humans might have a wide variety of presentations. In the majority of the cases, it is completely asymptomatic. A minority of the infected subjects develop symptoms, which range from fever, headache, gastrointestinal discomfort, skin rash, to serious neurological disorders.

The 2018 West Nile Virus (WNV) season in Europe was characterized by an earlier start and an exceptionally higher burden when compared to previous seasons [1,2]. Overall, in 2018, 1605 cases of WNV infections were reported in Europe. Italy was the most-affected country, accounting for 39% of the locally acquired infections. Overall, there was a 10.9-fold increase in incidence when compared to 2014–2017 seasons, and a 12-fold increase in comparison to 2017. Infection rates per 100,000 population increased from 0.1 (in 2017) to 1.0 in 2018. Overall, t577 human cases were reported in Italy; WNV neuroinvasive diseases (WNNV) were reported in 230 cases, WNV-attributed deaths were 42. The majority of the infections were from the Veneto, Emilia-Romagna, Lombardia, Piemonte, Sardegna and Friuli Venezia Giulia (FVG) regions [3]. In the FVG region, located in the Northeastern corner of the country, more than 60 WNV infections were reported, including 35 probable and 25 confirmed cases. Among these, nine cases presented as WNNV, and four had a lethal outcome.

The aim of this report is to describe the neurological presentation of seven patients admitted to University Hospital of Udine with a diagnosis of WNNV. We focus on two patients who presented with an atypical manifestation. Table 1 summarizes our clinical experience.

## 2. Case 1

An 87-year-old man was admitted to Cardiology Intensive Care Unit on August 2018 for non-ST-segment-elevation myocardial infarction. His prior history was remarkable for ischemic cardiomyopathy and arterial hypertension. No invasive procedure was performed during the hospitalization. On day 3, the patient presented abrupt onset of fever, meningeal syndrome, generalized stiffness and diffuse neuropathic pain. He was alert and oriented and showed no focal deficit. His blood exams showed leucopenia (3580/mmc white blood cells (WBC)), thrombocytopenia (47,000 platelets/µL) and elevation of liver enzymes (aspartate aminotransferase (AST) 95 UI/L, alanine aminotransferase (ALT) 80 UI/L). The cerebrospinal fluid (CSF) was clear and showed hypoglycorrhachia (25%), hyperproteinorrachia (637 g/L) and hypercellularity (85 cells/µL, 48% polymorphonuclear). Gram stain, culture and serologic tests for common neurotropic viruses were negative. Brain magnetic resonance imaging (MRI) showed no abnormalities. Laboratory diagnosis of WNV infection was accomplished through biologic fluids testing for WNV- specific IgG and IgM antibodies, and reverse-transcription polymerase chain reaction (rt-PCR). Patient n.1 was tested positive for WNV rt-PCR on serum and blood, and specific IgM and IgG were found in both CSF and serum. A 5-day course of intravenous immunoglobulin (IVIG) at 0.4 g/kg/day and high-dose steroid therapy (500 mg/day for 2 days followed by 1 mg/kg/day with progressive de-escalation) were administered. The patient’s general status slowly improved. On day 10, the patient experienced an abrupt neurologic deterioration; he was found unconscious, with a Glasgow Coma Scale (GCS) of 3. Any organic cause of encephalopathy was excluded; his blood tests, head computed tomography (CT) scan, electrocardiogram (ECG) and electroencephalography (EEG) were inconclusive. CSF was checked again and showed residual cellularity (24 cells/mmc, 60% lymphocytes, 30% polymorphs). The patient was intubated and admitted to the intensive care unit (ICU). A few hours later, he spontaneously recovered and was extubated. During the next 2 weeks of hospitalization, the patient manifested three other similar episodes of “intermittent coma”. A conservative management was performed. He experienced a partial recovery and was discharged.

## 3. Case 2

A 57-year-old man with an autoimmune glomerulonephritis (being treated with steroids and mycophenolate) presented to the emergency department, with a history of three days of fever, confusion, diplopia, opsoclonus, multifocal myoclonus and generalized tremor. Blood exams and CT scan were unremarkable. Lumbar puncture revealed normal opening pressure, and the CSF analysis identified 12 cells/µL (30% polymorphonuclear, 60% lymphocytes), protein count of 200 g/L and glucose count of 70 mg/dL. The EEG showed slow bilateral diffuse slow waves. A presumptive diagnosis of rhombencephalitis with opsoclonus-myoclonus syndrome was made, and an empirical therapy with ampicillin, ceftriaxone, acyclovir and dexamethasone was started. Contrast enhanced brain magnetic resonance imaging (MRI) revealed no acute process or abnormality. During the hospitalizations, the patient’s condition worsened. He progressively became comatose (GCS of 4) and required ICU admission and mechanical ventilation. The laboratory documented the presence of WNV-RNA in CSF, urine and blood samples, and WNV-specific immunoglobulin M (IgM) in blood. Therefore, high doses of IVIGs and steroids were administered. On day 4, fixed bilateral mydriasis appeared. A CT scan showed massive intraparenchymal hemorrhage associated with fourth ventricle compression and tonsillar herniation. The following day, the patient expired. Post-mortem macroscopic examination of the brain showed diffuse malacia.

## 4. Discussion

WNV transmission season 2018 has been characterized by an extremely high infection rate. Overall, 2083 autochthonous infections were reported in 2018 in Europe, with an increase of 7.2-fold when compared to 2017 [1,2].

WNV diagnosis is mainly formulated by detecting specific IgM and IgG antibodies directed towards WNV, or by testing for viral nucleic acid in CSF, tissue, blood, or other body fluid. Evidence of IgM in CSF is the cornerstone of the WNV diagnosis because IgM does not cross the blood brain barrier. Nevertheless, serology has some pitfalls: IgM remains detectable for 2 to 12 months. Furthermore, the usual serological enzyme-linked immunosorbent assay (ELISA) may cross-react with other flavivirus infections or vaccination. There is a great variety of serological methods, but none of these provide a definitive diagnosis.

Nevertheless, any positive result requires a confirmation with more specific tests. Thus, when IgM are found positive, seroconversion (by subsequent convalescent sample testing, hemagglutination inhibition, IgG ELISA, or plaque reduction neutralization assay) should be demonstrated [4].

However, IgM and IgG testing might be insufficient in some cases. Antibodies might not be present during the window period, as to cause a delay in the diagnosis. Furthermore, special categories, such as immunocompromised patients, might not be able to mount an adequate serological response. In this setting, molecular testing for WNV could be useful; however it is known that viremia is generally rapidly vanishing, and lasts between 3 and 14 days. Prolonged viremia has been reported in immuno-compromised patients [5].

Several studies demonstrated that WNV RNA determination in whole blood shows higher reliability compared to other samples [6,7]. Detection of WNV in urine is not included in the current case definition of WNV disease postulated by the European Centers for Disease Control. Although WNV viral load in CSF and blood is usually transient, shedding in the urine can continue for longer periods after the acute phase [8]. Indeed, the US Centers for Disease Control guidelines have included the detection of RNA in body fluids other than blood and LCR, including viruria, in the diagnostic criteria for WNV diagnosis confirmation [9].

Furthermore, pathology may reveal peculiar histological pictures. Autopsy reports have shown perivascular lymphoplasmacytic infiltrates, both in leptomeninges and parenchyma, microglial nodules or acute neuronal necrosis. Inflammation generally involves deep nuclei, the cerebellum and brainstem, with neuronal loss and neuronophagia. In our cases, WNV was present in brain matter, a finding that suggests a strong correlation between its neurotropism with the distribution of the cerebral lesions [10].

Most WNV infections are usually asymptomatic; 30% of cases present with a febrile illness characterized by mild symptoms that typically last up to a week. Less than 1% of infections present with central nervous system involvement [11]. We reported two WNV cases consistent with severe brain stem involvement. Although there was no electrophysiological or neuroradiology evidence, both patients presented symptoms due to brainstem and basal ganglia damage. Case 1 showed neuropsychiatric disorder and loss of consciousness with no apparent organic explanation. Case 2 presented unique findings consistent with opsoclonus-myoclonus syndrome (OPS) complicated with a massive hemorrhagic stroke. To our knowledge, 14 cases of WNNV-related OPS have been reported to date [12,13]. The majority of the cases were reported among patients affected by neoplasm or immune system deficit, as well as our case. Confusion and delirium, which were the main presenting symptoms in patient n.2, were reported in 30% of the cases, usually after hospital admission. Overall, no specific MRI findings were described in WNV associated-OPS. However, brainstem infectious involvement has been speculated to cause secondary cortical hypoperfusion and subsequent dysfunction [14].

Overall, WNV-associated CNS vascular events are rare [12,15,16]. The main hypothesis regarding the pathophysiology of these events is an inflammatory vasculopathy secondary to WNV CNS invasion. It has been suggested that WNV reaches CNS through two different routes: spreading within the axons of peripheral nerves to CNS, and via the blood system. A number of in vitro analyses demonstrated that, similarly to other flaviviruses, WNV can directly infect the endothelial cells. Once penetrated in the CNS, WNV elicit complex immunitary responses, which involve both intrinsic CNS immune cells (microglia and astrocytes) and extrinsic defenses (T cells, B cells, and monocytes/macrophages), thereby, leading to cytokines release, perivascular inflammation, and neuronal proliferation [17,18,19]. This could provide an explanation of the WNV-related vasculitis.

Treatment for WNV infection is mainly supportive. No therapy has been proven useful in a randomized controlled trial. Few reports and preclinical studies suggest the utility of immunomodulation through IVIG, plasmapheresis, WNV-specific neutralizing antibodies, corticosteroids, ribavirin, interferon or other RNA inhibitors [14].

## 5. Conclusions

The 2018 West Nile outbreak in FVG has been characterized by extraordinary high rates of WNV infections. The atypicality of the presentation of some of the WNNV forms highlights the necessity to stay vigilant and suspect the diagnosis when confronted with neurological symptoms. Given that no treatment has shown real efficacy, there is no consensus about the appropriate treatment. With regards to all the mosquito-borne infections, prevention measures, including personal protection and mosquito control programs, are necessary.

## Figures and Tables

**Table 1:** West Nile neuroinvasive disease: A single center experience.

Sex, Age (Years)	Previous Clinical History	Date of Infection	Clinical Form	Diagnosis *	Therapy	Outcome and/or Sequelae
Male, 33	Epilepsy	July 2018	VII cranial nerve palsy	Serology (IgM) in serum and LCR	Steroid	Healed
Female, 15	Previously healthy	July 2018	Fever, cephalea, Parkinsonism, left arm strength deficit, tongue fasciculation	Serology (IgM and IgG) in CSF	None	Healed
Male, 87	Ischemic cardiomyopathy	August 2018	Fever, meningismus and neuropathic pain, limbs waxy flexibility and catatonic states.	rt-PCR in both urine and CSF; serology (IgM and IgG) in both CSF and serum	IVIG and steroids	Residual disability, depression
Male, 56	Renal transplant, previous CMV and VZV reactivation	August 2018	Fever, confusion, meningismus	Serology (IgM) in serum; rt-PCR in urine	IVIG and steroids	Healed
Male, 57	Autoimmune glomerulonephritis in immunosuppressive treatment	September 2018	Rhombencephalitis, coma, intracranial hemorrhage	Serology (IgM) in serum; rt-PCR in CSF, urine and blood	IVIG and steroids	Death
Female, 78	Diabetes, hypertension, non-Hodgkin lymphoma	October 2018	Confusion, stupor, coma	rt-PCR in CSF, urine and blood		Death
Female, 77	B chronic lymphocytic leukemia, hypogammaglobulinaemia, hypertension, diabetes, previous CMV reactivation	November 2018	Fever, confusion, 4 limbs hyposthenia, acute kidney injury, coma	rt-PCR in CSF and urine	IVIG and steroids	Partial improvement, residual disability

Abbreviations—rt-PCR: reverse-transcription polymerase chain reaction, IgM: immunoglobulin M, IgG; immunoglobulin G; IVIG: intravenous immunoglobulin; CSF: cerebrospinal fluid; CMV: cytomegalovirus; VZV: varicella zoster virus. * Only positive results are reported.
